# The projection of Chinese University online image and social media engagement based on Bayesian model

**DOI:** 10.1371/journal.pone.0300625

**Published:** 2024-04-16

**Authors:** Dongmei Xia, Pengfei Zhao, Ji Wang, Yingji Li

**Affiliations:** 1 School of Education Science, Xinjiang Normal University, Wulumuqi, China; 2 Vocational Ability Development Research Center, Chongqing Institute of Engineering, Chongqing, China; 3 School of Humanities and Management, Yunnan University of Chinese Medicine, Kunming, China; Zhejiang Gongshang University, CHINA

## Abstract

Social media platforms provide the public with a forum for interaction and communication with tourism destinations, playing a significant role in the shaping and dissemination of destination images. Similarly, social media plays a vital role in the construction and propagation of online images for higher education institutions. For instance, indicators such as likes, shares, and visits on Weibo can serve as measures of public engagement with university social media. To reveal the triggering rules of social media engagement by projected images of destinations and related factors, this paper builds a Bayesian model using data from posts and interactions on the official Sina Weibo account of a Chinese university from 2018 to 2023. This model simulates to infer the optimal decisions that trigger university social media engagement.

## 1. Introduction

With the advent of the social media era, the marketing focus of destination management organizations has gradually shifted from traditional offline channels to the online space. The dissemination of information containing marketing strategies and media materials on social media platforms has become an essential means for destination management organizations to establish, maintain, and enhance the attractiveness of tourism destinations [1, 2]. In recent years, universities have garnered attention from both academia and industry in terms of online marketing and media image building. Universities have transitioned from traditional "ivory tower" images to more accessible and "cute" images in the public eye, gaining a large number of followers on social media platforms and sparking several viral trends. However, the appearance of highly popular topics with high attention and high dissemination on social media platforms also contains an element of randomness and uncertainty. Faced with the massive amount of information and complex communication structures on social media platforms, there is still a need to summarize the regularities in triggering public high-interaction participation through destination image projection. Additionally, universities possess rich and multidimensional destination images and play important roles in cultural heritage and public services. Effectively projecting destination images and establishing good interactive communication with the public to continually expand the scope and influence of information dissemination remain crucial challenges for university destination management organizations.

Participation and interaction are crucial stages in which the public constructs and perceives destination images on social media platforms. They can stimulate changes in the willingness to apply for university admission among university or high school students [3, 4]. Previous studies have identified factors influencing public participation on social media platforms, including content publication, timing of publication, language characteristics, and interaction features [5], using statistical methods such as structural equation modeling, multiple regression, and difference testing. These studies have preliminarily explored many factors affecting social media participation and their interactions. However, few studies can simulate and predict the degree of social media participation. Furthermore, existing research has not revealed which factors or combinations of factors can achieve optimal social media participation effects, making it difficult to answer the question of "how " destination management organizations should act on social media platforms.

This study uses the official Sina Weibo account of a Chinese university as an example, aiming to answer how educational institutions should project destination images on social media platforms to maximize the effect of social media engagement. First, we outline the basic characteristics of post frequency and social media engagement on the university’s official Sina Weibo, identifying the factors influencing social media participation. Second, we construct a Bayesian network model for social media engagement, simulating the fundamental principles of how destination image projection triggers social media participation. Finally, through model adjustment, we explore the optimal scenarios for maximizing social media engagement and achieve predictions of social media engagement effects under different decision-making scenarios.

## 2. Literature review

### 2.1 Destination image projection

Since the concept of destination image was introduced in 1971, it has been a research hotspot and focus in the field of tourism studies [6]. Destination image refers to the overall impression formed by the various tourism products and elements of a tourist destination [7]. The projected image is mainly initiated and conveyed by DMOs, while the perceived image is oriented towards tourists. Because consumers translate the information conveyed by destinations through their self-perception, their response to the brand may differ from expectations. Cultivating a close relationship between tourists and destinations on social media platforms has become the key to successfully projecting and marketing a destination image [8, 9]. Research in the field of brand marketing has shown that the information released by marketing organizations can stimulate consumers’ willingness to interact on social media platforms [10], establish close relationships between consumers and brands [11], and shape consumers’ emotional attachment [12, 13], loyalty [14], recommendation, and word-of-mouth behavior toward the brand [15]. In other words, the information released by destination marketing organizations on social media platforms not only shapes the image of tourist destinations but also utilizes network technology to realize interaction with tourists or potential tourists [16], and develop and maintain the relationship between tourists or potential tourists and tourist destinations.

In recent years, destination branding has been gradually applied to the field of university brand marketing [17]. Concerning the choice of university degrees and institutions themselves, students are no longer passive consumers [18]. In the digital age, the interaction between universities and potential applicants online has an impact on the decision-making process of applicants [19, 20]. Research has shown that potential applicants use a wide range of digital media, such as the social media platform TikTok [21], dedicated applications such as ChaseDream.com [22], as well as traditional websites and search engines. This means that discerning applicants use a variety of different digital resources to obtain decision-making information [19]. Especially during the COVID-19 pandemic, students’ perceived value in digital media has a positive impact on their destination selection intentions. It can be said that in the Internet society, the digital marketing abilities and standards of higher education institutions have never been so important in influencing the decision-making process of applicants [23].

Researchers have found that understanding and predicting the participation behavior of social media users plays an important role in destination image projection and marketing [24, 25]. With the rise of social media platforms represented by Weibo in Chinese universities, destination management organizations, including Tsinghua University, Peking University, Fudan University, Wuhan University, and others, have registered official accounts on social media platforms to communicate and interact with social media users, to construct and maintain close connections between destinations and university students, alumni, or high school students [26]. However, there is limited research focusing on the relationship between the destination projection image of universities and social media participation, which is only limited to identifying differences in the number of forwarding, commenting, and liking caused by destination projection images [27], and has not yet addressed how to use university destination projection image to enhance social media participation.

### 2.2 Social media participation and its influencing factors

The higher education environment is also undergoing a period of significant change [28]. Viewing students as consumers is one of the changes observed in higher education [29, 30]. Scholars found that watching time can greatly affect the promotion and admission of higher education institutions by analyzing the data of the UhamkaTV YouTube channel [31]. Therefore, social media is an important medium for connecting students, teachers, parents, alumni, and other stakeholders. Social media participation is a direct manifestation of network adhesion of university applicants.

Participation is a complex and multidimensional concept that includes various forms of cognition, emotion, and behavior [32]. On social media platforms, participation is usually measured at the behavioral level, manifested as sharing, commenting, or liking [33, 34]. However, users’ behaviors such as sharing, commenting, and liking have different behavioral logics, and simply adding up these numerical values does not reflect their underlying behavioral differences. Among them, liking represents the most basic and simplest form of social media participation [33, 35]. Commenting involves users providing feedback in semi-public online communities created by posts, expressed through text, images, and other forms [36]. Sharing involves users sharing post content with friends and other social media users, driving the dissemination and diffusion of original post content, and showcasing self-image and identity [37]. In other words, the content released by destination management organizations and the resulting likes, comments, and reposts form a semi-public network community, achieving the function of identifying and cultivating potential tourists, and influencing the dissemination and spread of the destination projection image of universities.

Previous research has found that factors such as account attributes, posting content, affective representations, and posting time collectively influence users’ social media engagement behaviors [38]. Although the specific content of posting varies in different research scenarios, it shows that highly interactive information can trigger more attention and participation behavior [39]. Therefore, university microblogs can enhance the interactivity of messages and guide social media users to participate in online activities by providing links to websites, designing sweepstakes contests, and setting up questions [40]. Some scholars classify online brand-related activities of social media users into three types: consumption, contribution, and creation [41]. Based on this, this study classifies the marketing activities used by message publishers into consumption guidance, contribution guidance, and creation guidance, to portray the types of interactive guidance used in messages. Consumption guidance refers to guiding social media users to view and download relevant content; contribution guidance refers to guiding social media users to comment and retweet relevant content; and creation guidance refers to guiding social media users to design and publish relevant content.

In addition to the content of the message, the emotional characteristics presented in the message also affect social media engagement [42]. Research on the dissemination of university admissions information found that messages containing emotions increased social media users’ liking and retweeting behavior, but did not affect the number of comments [43]. Recent research has shown that messages containing emotional content on social media platforms are more likely to go viral, have a greater reach [44], and contribute to a stronger connection between social media users and the brand [39, 45]. At the same time, the destination image projected by universities is not only a display and introduction of destination attributes but also a way to evoke an emotional experience and create a positive destination image [46]. In addition, the level of social media engagement is also related to the time of posting [47], the date of posting [43], and seasonality [48]. Research on Facebook has found that social media users post more comments on weekdays, but do not generate more likes or retweets [49]. Some scholars have also found that the date of posting does not affect the number of likes and comments [47]. Travel information posted on weekends can achieve higher social media engagement. It can be seen that there is still no unanimous conclusion on when to post information to form the best social media interaction and marketing effect.

Based on existing research, this article focuses on four factors that influence social media participation in official school Weibo platforms, namely destination image projection, interactive strategies, emotional representation, and time frame. It distinguishes the different implications of reposts, comments, and likes in public social media participation, simulates the impact of different factors and factor combinations on these three social media participation behaviors, and explores a guideable and predictable optimal path for university destination image projection.

## 3. Data source and research methodology

### 3.1 Research design

This study uses PyCharm Community Edition (2023.1.2) as the tool for data collection. PyCharm is a powerful Integrated Development Environment (IDE) for Python, providing numerous features and tools to facilitate developers in coding, debugging, and testing. Although PyCharm itself does not have specific functions directly used for web scraping or data extraction, it provides strong support for Python interpreters and libraries that can be utilized to implement these functions. We import the ’requests’ and ’pandas’ libraries in PyCharm, and use Python code to scrape data. The Python code we use for data scraping is shown in Table 1:

**Table 1:** The Python code used for scraping Sina Weibo.

import requests import pandas as pd def crawl_weibo(username, limit = 1): """ retrieve all Weibo posts made by a specific account """ api = ’https://m.weibo.cn/api/container/getIndex’ data = {’text’: [], ’year’: [], ’month’: [], ’time’: [], ’reposts_count’: [], ’comments_count’: [], ’attitudes_count’: []} since_id = None # Starting Weibo ID while True: params = { ’type’: ’uid’, ’value’: username, ’containerid’: f’107603{username}’, ’since_id’: since_id, } headers = { ’User-Agent’: ’Mozilla/5.0 (Windows NT 10.0; Win64; x64) AppleWebKit/537.36 (KHTML, like Gecko) Chrome/95.0.4638.54 Safari/537.36’, ’Referer’: f’https://m.weibo.cn/u/{username}’, ’Cookie’: ’SUB = _2A25NwWQBDeRhGeBG7FUU9S7EyDiIHXVv1fSPrDV6PUJbkdAKLUumkW1NSFeNU4-BZPxnfxYUL0PUuoLbcoIfhWuk;’ } res = requests.get(api, headers = headers, params = params) cards = res.json().get(’data’, {}).get(’cards’, []) if not cards: # reached the last page, exiting the loop break else: for card in cards: if card.get(’card_type’) = = 9: weibo = card[’mblog’] created_at = pd.to_datetime(weibo[’created_at’]) text = weibo.get(’text’) if not text: text = weibo.get(’longText’, {}).get(’longTextContent’, ’’) year = created_at.year month = created_at.month time_ = created_at.time().strftime(’%H:%M:%S’) reposts_count = weibo.get(’reposts_count’) comments_count = weibo.get(’comments_count’) attitudes_count = weibo.get(’attitudes_count’) data[’text’].append(text) data[’year’].append(year) data[’month’].append(month) data[’time’].append(time_) data[’reposts_count’].append(reposts_count) data[’comments_count’].append(comments_count) data[’attitudes_count’].append(attitudes_count) if len(data[’text’]) > = limit: break since_id = cards[–1].get(’mblog’, {}).get(’id’) # Start the next request from this Weibo ID if len(data[’text’]) > = limit: break return pd.DataFrame(data) username = ’1676317545’ # Weibo user ID df = crawl_weibo(username, limit = 10000) # Set the maximum number of crawls to 3000 df.to_excel(’weibo_data_QH1.xlsx’, index = False) # Save as an Excel file

This paper investigates the image projection and social media engagement of universities, focusing on Tsinghua University in China. As one of the most renowned institutions in China, Tsinghua University is representative of this study. Its official Weibo account frequently updates with a diverse range of content types. The data collected for our research comprises all posts made by Tsinghua University’s official Weibo account from 2018 to 2023. Our selection criterion was based solely on the timeline, disregarding data before 2018 due to its relative obsolescence and limited relevance to the current study. Additionally, because of platform updates, complete data retrieval for Weibo posts before 2018 is not feasible, which further justifies their exclusion.

During the data collection stage of our study, we followed the terms and conditions of the data source and respected the ethical norms of information collection. Our data collection and analysis were done using Python to ensure the reliability, validity, and integrity of the data. We implemented a series of strict measures to comply with the terms and conditions of the data source. For instance, we only collected data from publicly licensed resources, such as the official Sina Weibo of Tsinghua University. We made sure that all data collection processes followed relevant laws and regulations. Additionally, to protect the privacy of participants, we anonymized all collected data. We also respected the ethical norms of information collection by informing all participants about how their data would be used and obtaining their consent beforehand. We removed or replaced personally identifiable information to prevent any possible infringement. Furthermore, our research design, including sample selection, data collection strategy, and statistical analysis method, underwent review by the academic paper committee of Tsinghua University. They evaluated our research and agreed that it complied with all relevant academic and ethical standards.

### 3.2 Data processing

In this study, PyCharm Community Edition (2023.1.2) was used as the text mining tool to collect all posts published on Tsinghua University’s official Sina Weibo account from January 1, 2018, to August 29, 2023. A total of 6532 valid texts were included in the research scope, as shown in Table 2. It is important to note that during the COVID-19 pandemic period from December 2019 to December 7, 2022, social media provided a unique platform for universities, enabling the public to gain deeper insight into the universities’ public image and needs. Simultaneously, it influenced the public’s attitudes and behaviors toward universities [50].

**Table 2:** A university has published Weibo and received retweets over the years (2018–2023).

Index	2018	2019	2020	2021	2022	2023
Number of Weibo	945	1019	1335	1175	1317	669
Average Retweets	14	8	16	13	11	9
Average number of comments	54	62	51	63	75	45
Average number of likes	137	128	127	102	114	96

Improve that Weibo text content and comment content are not on the same page and are progressively related, a significant amount of duplicate Weibo text content is produced. Therefore, this study utilizes the duplicate data removal function built into Microsoft Office Excel 2013. The reference for deletion is the consistent point of praise, comments, and repost volume. At the same time, some redundant content not within the statistical term was also deleted. Special symbols were handled accordingly. Most special symbols and emojis in this study appear in the comments. To deal with this, we only read the data after the first colon when reading the data, ignoring the nickname of the publisher. On the other hand, the remaining special symbols are removed through Excel’s replace function and Python’s delete function. The original data, after the aforementioned processing and time-based sorting, is shown in Table 3.

**Table 3:** Display of some microblog text data after processing.

Serial number	original text	Processed text	semiscore	semilabel
**1**	【Let’s learn together! The First Lesson for Tsinghua University’s Freshman Class of 2023】 What did the first lesson for Tsinghua University’s freshman class of 2023 cover? How can students embark on a new phase of their lives as Tsinghua students with a new mindset? Qiu Yong, Secretary of the Party Committee of Tsinghua University, gave a lecture for the first day of school on the theme of "being a new era person who is capable of fulfilling the responsibilities of national rejuvenation with vigorous efforts and bravery." Check the link for more details↓ #BacktoSchool# The Secretary of Tsinghua University’s Party Committee, Qiu Yong, gave the first lesson to the undergraduate freshmen of the class of 2023.	This is a repetition of the previous Chinese text. Here is the translation again: "Let’s learn together! The First Lesson for Tsinghua University’s Freshman Class of 2023" What did the first lesson for Tsinghua University’s freshman class of 2023 cover? How can students embark on a new phase of their lives as Tsinghua students with a new mindset? Qiu Yong, Secretary of the Party Committee of Tsinghua University, gave a lecture for the first day of school on the theme of "being a new era person who is capable of fulfilling the responsibilities of national rejuvenation with vigorous efforts and bravery." Check the link for more details. #BacktoSchool# The Secretary of Tsinghua University’s Party Committee, Qiu Yong, gave the first lesson to the undergraduate freshmen of the class of 2023.	1	1
**2**	【Speech by President Wang Xiqin at the Opening Ceremony for Undergraduate Freshmen of 2023 at Tsinghua University | Diligence Leads to Progress, Step by Step】#OpeningCeremonyforUndergraduateFreshmen# #BacktoSchool# Click to view the speech by President Wang Xiqin at the Opening Ceremony for Undergraduate Freshmen of 2023 at Tsinghua University | Diligence Leads to Progress, Step by Step.	This is a repetition of the previous Chinese text. Here is the translation again: "Speech by President Wang Xiqin at the Opening Ceremony for Undergraduate Freshmen of 2023 at Tsinghua University | Diligence Leads to Progress, Step by Step" #OpeningCeremonyforUndergraduateFreshmen# #BacktoSchool# Click to view the speech by President Wang Xiqin at the Opening Ceremony for Undergraduate Freshmen of 2023 at Tsinghua University | Diligence Leads to Progress, Step by Step.	0.414085578	0
**3**	【Tsinghua Teacher Representative Tian Ling: Sweating and Harvesting Growth】#OpeningCeremonyforUndergraduateFreshmen# #BacktoSchool# Tsinghua Teacher Representative Tian Ling: Sweating and Harvesting Growth.	This is a repetition of the previous Chinese text. Here is the translation again: "Tsinghua Teacher Representative Tian Ling: Sweating and Harvesting Growth" #OpeningCeremonyforUndergraduateFreshmen# #BacktoSchool# Tsinghua Teacher Representative Tian Ling: Sweating and Harvesting Growth.	0.999999998	1
……				
**6532**	【Tsinghua Undergraduate Representative Xu Hui: Pursuing a Sense of Meaning in University Life】#OpeningCeremonyforUndergraduateFreshmen# #BacktoSchool# Tsinghua Undergraduate Representative Xu Hui: Pursuing a Sense of Meaning in University Life.	This is a repetition of the previous Chinese text. Here is the translation again: "Tsinghua Undergraduate Representative Xu Hui: Pursuing a Sense of Meaning in University Life" #OpeningCeremonyforUndergraduateFreshmen# #BacktoSchool# Tsinghua Undergraduate Representative Xu Hui: Pursuing a Sense of Meaning in University Life.	0.99999976	1

**Fig 1:**
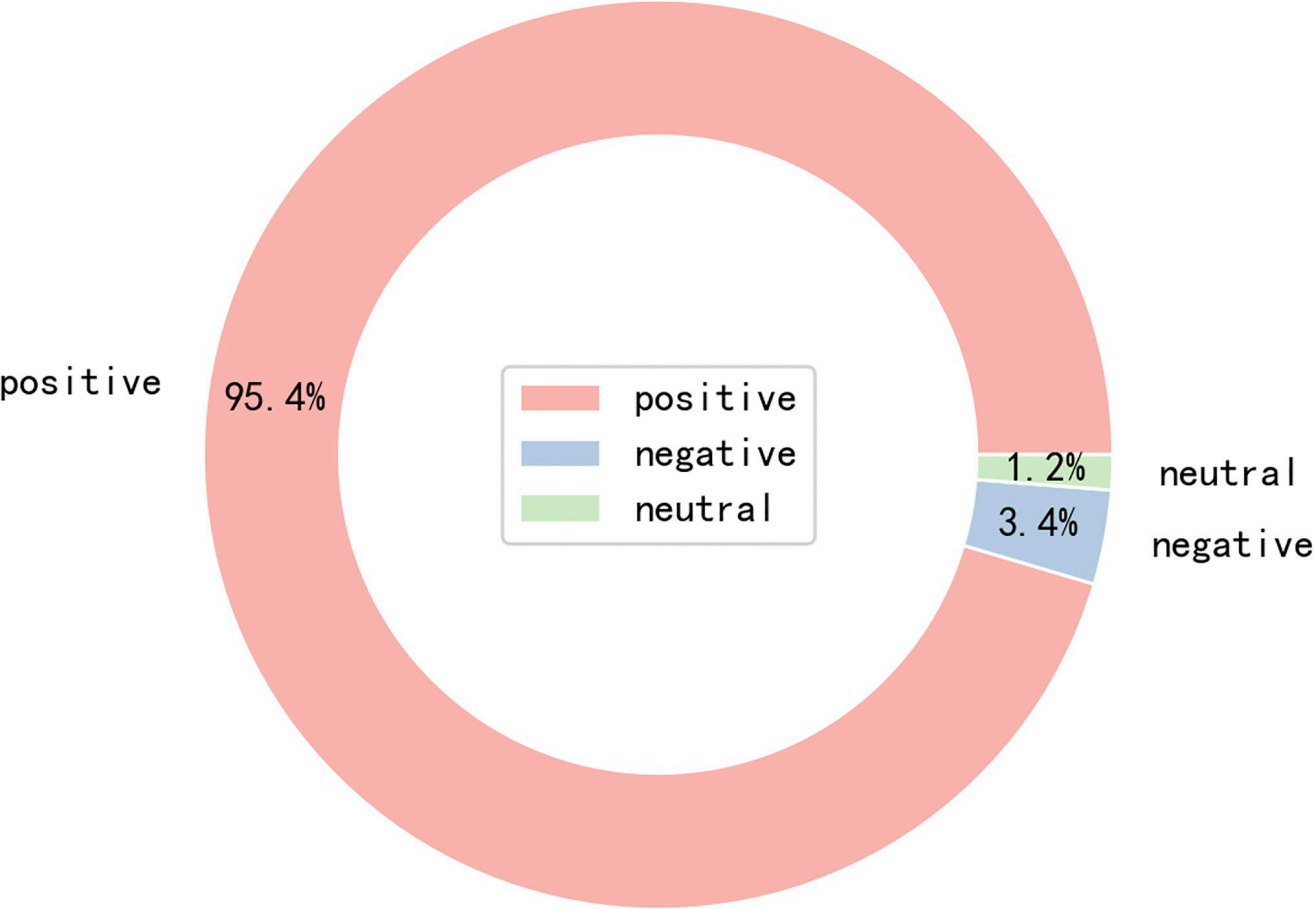
Emotion classification Pie chart of texts from official Weibo account of a Chinese University.

To conduct statistical analysis of hot topics, this study used the Jieba package (Chinese for “tostutter”, V 0.38) based on Python to segment the crawled post text string. In segmentation, stop words such as "的," "吗," and "和," which are non-substantive words, were removed using a stop word table. Finally, the combination of Python Snownlp and the sentiment dictionary was used to perform sentiment analysis on the segmented results, which was used as the basis for comprehensive analysis and calculation of the Weibo text sentiment value, as shown in Fig 1.

### 3.3 Variable selection

Based on existing research, this study focuses on four factors that influence social media participation: destination image projection, interactive strategies, emotional representation, and time frame. Destination image projection is divided into six dimensions: university buildings, natural landscapes, historical and cultural aspects, creative products, facilities and services, and student activities. Interactive strategies are categorized into two indicators: interactive guidance and mentions of others. Interactive guidance refers to how Weibo text guides social media users’ participation behavior and can be divided into four categories: no guidance, application guidance, contribution guidance, and collaboration guidance. Mentioning others refers to whether the Weibo text uses the "@" function to mention other social media users and is categorized as either present or absent.

Emotional representation is divided into positive emotions, negative emotions, and no emotions, and these categories are obtained as continuous data using Python. The time frame includes three indicators: season, date, and posting time. Seasons are categorized according to meteorological divisions, with March to May as spring, June to August as summer, September to November as autumn, and December to February of the following year as winter. Posting dates are divided into weekdays and weekends, considering a one-week cycle. Posting times are categorized into five time periods: 8:00–11:59, 12:00–13:59, 14:00–17:59, 18:00–22:59, and 23:00–7:59.

Social media engagement in this study comprises three indicators: retweets, comments, and likes. The retweets, comments, and likes data collected through web scraping are all continuous variables and are statistically significant for variance analysis and influence graph modeling. However, Bayesian models require nominal variables, so it was necessary to convert continuous variables into nominal ones. To achieve this, the Weibo posts were sorted based on the number of retweets, comments, and likes in descending order, and the top 20% of Weibo posts were categorized as high retweet, high comment, and high like Weibo posts, while the remaining Weibo posts were categorized as low retweet, low comment, and low like Weibo posts. These categories were used for constructing Bayesian models and statistical inferences.

### 3.4 Research methodology

#### 3.4.1 Research steps

The inference process for the optimal scenario in this study can be roughly divided into 5 steps. The first step involves collecting data from Weibo posts published by the university’s official Weibo account and encoding it. Six nominal variables are formed, representing the dimensions of destination image projection, interactive guidance, mentioning others, seasonality, date, and posting time. Emotional representation scores (positive emotions, negative emotions) are calculated, and both emotional representation and social media engagement data (retweets, comments, likes) are converted into nominal variables. Continuous variables are used for Bayesian model parameter determination and expected utility inference, while nominal variables are used for Bayesian model parameter determination and probability inference. The second step utilizes SPSS 23.0 software to conduct chi-squared tests and analysis of variance to identify factors influencing social media engagement. In the third step, based on existing research and the conclusions from step two, a Bayesian model is constructed using Netica V5.18 software. This involves parameter mathematical learning using the encoded Weibo data to determine Bayesian model parameters. Sensitivity analysis and probability inference are then carried out, targeting social media engagement as the goal node, to identify key factors and factor combinations influencing social media engagement with destination image projection. The fourth step extends the Bayesian model by incorporating decision nodes and value nodes to construct an influence diagram model based on the Weibo posting decision process. In the fifth step, considering the findings from the previous steps, the values of the influence diagram model nodes are adjusted. This allows for the simulation and calculation of the expected utility of social media engagement, resulting in the inference of the optimal path for inducing social media interaction through destination image projection.

In comparison to classical statistical methods, Bayesian methods, including Bayesian models and influence diagrams, have the ability to integrate multiple data sources, perform simulation inference, and provide graphical representations. They are effective at handling relationships between prior knowledge and new observational data, allowing for simulations under different scenarios, and offering an intuitive display of the probability changes for decision-making. Bayesian methods have been widely applied in fields such as organizational management, environmental management, risk management, and consumer behavior to predict engineering issues, risks, and consumer behavior probabilities. In recent years, scholars have increasingly applied Bayesian models to analyze student behavior and have made significant progress. Influence diagrams extend Bayesian models for probabilistic inference of discrete variables into predicting expected utility for decision alternatives. They capture not only decision alternatives and decision preferences but also compute the expected outcomes of different decision alternatives. Influence diagrams have been widely applied in fields such as risk decision-making and organizational decision-making. The application of this method in school management research has promoted innovative methods in the study of university network image projection and social media marketing.

#### 3.4.2 Bayesian modeling

Bayesian networks are graphical models based on probabilistic descriptions of dependencies between data variables, capable of probabilistic inference in the presence of multiple factors and uncertain information. A Bayesian model consists of a directed acyclic graph and a conditional probability table. The directed acyclic graph consists of nodes and directed arcs, where the nodes represent the variables and the directed arcs represent the influence of the parent nodes to the child nodes; the conditional probability table is used to represent the strength of the relationship between the nodes. The Bayesian formula is considered to be the basis of Bayesian modeling and can be expressed as:

$$P(X|Y)=\frac{P(Y|X)P(X)}{P(Y)}$$ (1)

In Eq (1), the P(X|Y) is the known event Y occurring at the time of X the probability of occurrence, and P(X|Y) is the probability of X the conditional probability of the occurrence of Y of the conditional probability. Two random variables X and Y The joint distribution of can be expressed as:

P(X,Y)=P(X)P(Y|X) (2)

In Eq (2), the P(X) is called the a priori probability and P(Y|X) is the posteriori probability. The a priori probability is the probability that the factor occurs independently and separately from the other factors. The posteriori probability is the probability of the factor occurring given the probability of the antecedent factor. The complexity of the probabilistic model can be reduced by combining the chain rule, then The joint distribution of the individual variables is:

$$P(X_{1},X_{2},\ldots ,X_{n})=\prod_{i=1}^{n}P(X_{1}|X_{pa(i)})$$ (3)

Set S to be a network structure containing X the structure of a network of variables, where $X=\{X_{1},X_{2},\ldots ,X_{n}\}$, P is the set of local probability distributions associated with each variable, and *X*_1_ denotes a variable node, and *X_pa(i)_* denotes the parent node of the variable node in the network structure S. According to the above formulas, the conditional probability table for each node can be calculated and the probability inference for each node of the Bayesian network model can be performed according to the Bayesian formula.

## 4. Data analysis and research results

### 4.1 Number of Weibo posts published by a certain university’s official Weibo account and its influencing factors

We conducted a statistical analysis of the number of Weibo posts published by a certain university’s official Weibo account from 2018 to 2023, considering different dimensions of destination image. We also examined their combinations with interactive strategies, emotional representation, and temporal frameworks, followed by conducting chi-square tests (Table 4). The results reveal that the university’s official Weibo account employs distinct interactive strategies, emotional representations, and differentiated posting schedules when projecting different dimensions of destination image.

**Table 4:** Cross-tabulation of the difference in the number of microblogs between projected images and interaction strategies, affective representations, and time frames at a university in China.

Variables		Level	Projected destination image						
			School buildings(bu)	School natural landscape(nl)	School history and culture(hc)	School creative products (cc)	Student activities(sa)	School facilities services(sf)	Cardina x^2^ (sig.)
Interactive strategies	Interactive guidance	No guidance	1024	552	2416	58	509	96	12.000^a^(0.213)
		Application guidance	118	89	239	17	236	96	
		Contribution guidance	89	147	254	5	86	77	
		Collaboration guidance	59	21	134	13	113	84	
	@others	Yes	257	215	863	18	362	98	2.000^a^(0.157)
		No	1033	594	2180	75	582	255	
Emotional representation	Emotion	Positive emotion	628	386	1796	33	475	186	6.000^a^(0.199)
		Negative emotion	0	0	0	0	0	0	
		Not have	662	423	1247	60	469	167	
Time frame	Seasons	Spring(march-may)	417	209	715	26	293	109	12.000^a^(0.213)
		Summer (june-august)	193	179	771	20	172	63	
		Fall(september-october)	462	230	798	28	315	122	
		Winter(december-february)	218	191	759	19	164	59	
	Post date	Workdays	1020	601	2360	79	812	287	2.000^a^(0.157)
		Weekends	270	208	683	14	132	66	
	Post time	8:00–11:59	512	321	690	0	459	128	20.000^a^(0.220)
		12:00–13:59	214	69	271	17	122	43	
		14:00–17:59	350	271	1165	63	248	116	
		18:00–22:59	199	145	858	10	112	63	
		23:00–7:59	15	3	59	3	3	3	
Total			1290	809	3043	93	944	353	

From the perspective of destination image dimensions, it is noteworthy that the university’s Weibo account predominantly posts content related to the historical and cultural dimension, which accounts for over 47% of all Weibo posts. Following closely are the dimensions of school buildings and student activities, both exceeding 14% of the total Weibo posts. Conversely, the dimensions related to school facilities and services, among others, have fewer posts.

Regarding interactive strategies, only 29% of the Weibo posts employ interactive guidance, and merely 5% of the posts mention others. Notably, the dimensions of university activities and cultural events utilize more application and cooperation guidance strategies (For example: The enrollment has commenced this year. If you are passionate about the destiny of Earth and humanity, have a strong academic interest, and demonstrate outstanding potential for innovation, we welcome you to join Xiuzhong Academy!), often mentioning other social media users(For example: On the afternoon of June 19, Zhu Bangfen, a member of the Chinese Academy of Sciences and the chief professor of Mathematics and Physics at Tsinghua University, will provide an in-depth explanation on all aspects of training talents in Mathematics and Physics. #CollegeEntranceExamTogether #UniversityWeiboCloudEnrollment). In contrast, other dimensions tend to use contribution guidance more frequently, while employing fewer interactive strategies overall.

In terms of emotional representation, approximately 53.6% of the Weibo posts incorporate positive emotional vocabulary (For example: As we step into this campus, we also step into each other’s comfort zones. At the beginning of our freshman year, referring to "returning to the dorm" as "going home" was merely for convenience or perhaps an attempt to create a sense of belonging through language.), albeit with relatively lower positive emotional scores. Remarkably, no negative emotional vocabulary is present in these posts.

When examining the temporal framework, the university’s official Weibo account tends to post more Weibo content during the autumn enrollment season compared to the spring enrollment season, especially within the dimensions of school buildings (For example: [A letter from winter! #Beijing’sFirstSnow, a glance at Tsinghua campus] Unseen in the sky, someone plays the flute, casting down blossoms of snow over the world. The first snow of winter has quietly arrived, with snowflakes gently covering every corner of the Tsinghua campus.) and natural landscapes (For example: [Snowfall brings a new understanding; flying snowflakes fill Tsinghua] The north wind is desolate, and all things are in deep slumber; the winter wind is sharp, snowflakes are fluttering, and majestic Tsinghua stands starkly in pure white. The chill carves out jade bones and ice skin, tracing the contours of the wind. With everything sharing the same hue, we appreciate both past and present in their silver-dressed purity.). Furthermore, during weekdays, there is a slightly higher volume of Weibo posts compared to weekends, particularly within the dimensions of school facilities (For example: November 1, 10:33 [Old Library, Rebooted!] With history to the left and right, its grandeur stands tall. The mottled sunlight and soft indoor lighting illuminate the tranquility of time; the smell of ink on book pages emanates a historical aura. Come join us at #TsinghuaUniversityOldLibrary, and listen to the stories of the old library amidst the rustling sound of turning pages.) and student activities (For example: September 25, 09:35 [#TsinghuaUniversity2023SummerSocialPractice Exploring One Belt, One Road Towards Mutual Progress] Recently, the "Global Competency Overseas Practice Course" team from Tsinghua University visited Kenya and Rwanda. They went deep into the East African plateau, where they appreciated the cultural, social, and natural landscapes of both countries in their exploration of the "One Belt, One Road" initiative, gaining insights into their historical development, current status, and future prospects.). The time slot between 8:00 AM to 11:59 AM sees the highest number of Weibo posts, with an emphasis on the historical and cultural dimension. Meanwhile, the time slots of 14:00 PM to 17:59 PM witness an increase in Weibo posts related to school facilities and services, student activities, and other dimensions. However, during the time slots of 12:00 PM to 13:59 PM, 18:00 PM to 22:59 PM, and 23:00 PM to 7:59 AM (non-working hours), the university’s official Weibo account posts relatively fewer Weibo entries.

### 4.2 Social media engagement triggered by official university Weibo and its influencing factors

The number of reposts, comments, and likes on official university Weibo posts from 2018 to 2023 follows a power-law distribution, characterized by a small number of highly popular posts with a substantial amount of interactions and a large number of posts with very few interactions, failing to achieve high levels of social media engagement. According to the Pareto principle, the top 20% of posts by reposts account for 98.27% of the total reposts, the top 20% of posts by comments account for 90.20% of the total comments, and the top 20% of posts by likes account for 85.41% of the total likes. This indicates that social media engagement on official university Weibo is concentrated within a small number of popular posts, generating a significant amount of reposts, comments, and likes, thus exerting a considerable societal influence. However, a large number of posts, particularly those related to campus activities and cultural dimensions, have failed to generate significant social media engagement.

An analysis of the mean differences in reposts, comments, and likes on official university Weibo posts was conducted across different factors, including social media image projection dimensions, interactive strategies, emotional representation, and time frames (Table 5). The results indicate that different dimensions of social media image projection, types of interactive guidance, seasonality, and posting time significantly influence the number of reposts, comments, and likes on official university Weibo posts. Posts related to school creative products achieved the highest number of reposts, significantly surpassing posts from other dimensions. Posts related to student activities and school natural landscapes triggered higher levels of comments and likes. Posting time had an impact on comments and likes, while mentioning others only affected reposts. Positive emotions only affected comments and likes, whereas negative emotions did not significantly influence reposts, comments, or likes on official university Weibo posts.

**Table 5:** Differential analysis of the number of retweets and likes of the official blog of a university in China under the influence of different.

Variables	Levels	Average number of forwards	Average number of comments	Average number of likes
Projecting an image of a destination	BU	5.04	8.00	112.00
	NL	6.00	13.00	133.00
	HC	5.00	12.00	142.00
	CC	12.00	15.00	213.00
	SA	58.00	52.00	313.00
	SF	7.00	27.59	184.85
Interaction guide	No guidance	3.00	5.00	143.00
	Application guidance	12.00	26.00	156.00
	Contribution guidance	43.00	55.00	212.00
	Collaboration guidance	78.11	88.30	319.66
@Others	Yes	36.43	38.00	211.00
	No	4.00	10.32	144.56
Emotion	Positive emotion	21.64	25.00	223.00
	Negative emotion	0.00	0.00	0.00
	No emotion	3.00	9.90	93.57
Seasons	Spring	15.00	20.00	169.00
	Summe	9.00	14.00	152.00
	Fall	16.00	21.00	173.00
	Winter	10.30	15.30	152.51
Post date	Workdays	14.30	20.00	175.00
	Weekends	8.12	10.49	117.91
Post time	8:00–11:59	8.00	12.00	63.00
	12:00–13:59	18.00	23.00	291.00
	14:00–17:59	12.00	18.00	130.00
	18:00–22:59	20.04	25.00	306.00
	23:00–7:59	5.00	9.52	63.93

### 4.3 Bayesian model of social media engagement in official university Weibo

#### 4.3.1 Construction of the Bayesian model and sensitivity analysis

**Fig 2:**
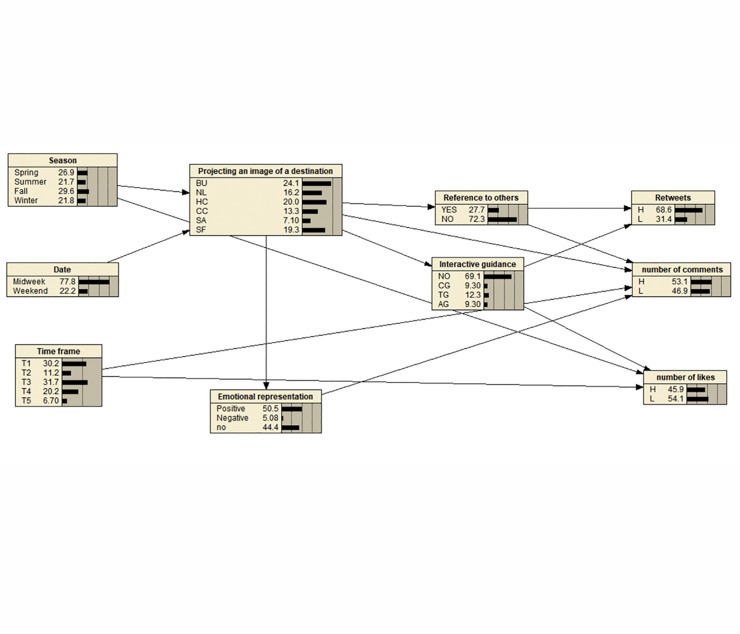
Bayesian model of social media engagement triggered by tourism destination image projection. **Note:** BU: School Buildings, NL: School Natural Landscape, HC: School History and Culture, CC: School Cultural and Creative Products, SA: Student Activities, SF: School Facilities and Services, NO: No Guidance, CG: Collaborative Guidance, TG: Contribution Guidance, AG: Application Guidance.

Based on existing research and the results of the statistical tests mentioned above, we constructed a Bayesian model of social media engagement triggered by the projection of destination images on official university Weibo. The direction of the connections follows the decision sequence during Weibo posting and has been confirmed for consistency by the three authors of this paper (Fig 2). A total of 2,095 data points containing eight variables, including social media image projection, interactive guidance, mentioning others, positive emotion, negative emotion, season, date, and posting time, were imported into Netica V5.18. We calculated the parameters of the Bayesian model using a parameter learning method. The target nodes were set as reposts, comments, and likes. Sensitivity analysis was conducted to assess how changes in the target nodes’ probabilities were influenced by changes in various factors or combinations of factors, as indicated by the color depth of nodes in the model.

Sensitivity analysis revealed the impact of changes in node probabilities on the probabilities of target nodes, helping to identify critical nodes affecting Bayesian inference results. The results showed that, except for negative emotions, adjusting the levels of various factors would affect social media engagement on official university Weibo. Among them, the posting time, image projection dimension, and interactive guidance type were factors with relatively high sensitivity. Small changes in the probabilities of these three nodes would lead to significant changes in the probability of social media engagement.

#### 4.3.2 Probability inference of university social media engagement scenarios

The goal of official university Weibo in projecting social media images is to trigger high levels of social media engagement, thereby increasing dissemination efficiency and social influence. Therefore, in this study, we set the states of the three nodes, reposts, comments, and likes, as high. We calculated the posterior probabilities of other nodes in the Bayesian model and revealed the direction and strength of the impact of various factors on social media engagement by observing changes in posterior probabilities (Table 6).

**Table 6:** Posterior probability changes of various influential factors in different scenarios of social media engagement.

Variables	Levels	Original probability(%)	Posterior probability (%)		
			Retweets = high	Comments = high	Likes Volume-High
Projecting an image of a destination	BU	0.24	56.4	60.2	44.7
	NL	0.16	56.8	45	45.4
	HC	0.20	56.9	47.8	45.7
	CC	0.13	56.5	43.1	48.1
	SA	0.07	56.7	49.9	45.8
	SF	0.19	56.9	57.1	42.9
Interactive guidance	NO	0.69	59.2	51.7	44.9
	CG	0.09	41.1	51.9	45
	TG	0.12	54.2	51.3	47.2
	AG	0.09	57.2	51.9	52.3
Reference to others	YES	0.28	29.9	48.6	45.9
	NO	0.72	83.4	54.8	45.9
Emotional	Positive	0.51	56.7	50.2	45.9
	Negative	0.05	56.7	49.8	45.9
	No	0.44	56.7	53.6	45.9
Season	Spring	0.27	68.6	54.5	42.8
	Summer	0.22	56.7	49.5	51.4
	Fall	0.30	56.7	51.7	48.4
	Winter	0.22	56.7	50.8	40.8
Date	Midweek	0.78	56.7	52	45.9
	Weekend	0.22	56.7	50.4	45.9
Time frame	T1	0.30	56.7	50.5	34.3
	T2	0.11	56.7	56.3	52.2
	T3	0.32	56.7	53.6	43.7
	T4	0.20	56.7	48.2	59.9
	T5	0.07	56.7	54.7	55.7

The results show that the posterior probability of school creative product dimensions and high positive sentiment increased significantly in both the high retweet, high comment, and high like scenarios. The posteriori probabilities for the spring school season increased more in the high retweet and high like scenarios, and the contribution guidance and creation guidance increased more in the high comment scenarios. From the results of the posterior probability changes, it can be seen that school’s natural landscape dimension, using interaction strategies and high positive emotions are the key to triggering high levels of social media engagement, while different interaction strategies can lead to the formation of different types of social media engagement, and tweets posted in the spring enrollment season and non-study time are more likely to trigger high social media engagement.

## 5. Conclusions and outlook

### 5.1 Conclusion and discussion

This study uses the number of forwards, comments, and likes as indicators to evaluate public social media engagement. It constructs two hierarchical decision reference models: a Bayesian model and an influence diagram. The study simulates and predicts the optimal scenarios and expected effects of information dissemination by destination management organizations, bridging the complex relationship between destination image projection and social media engagement.

In terms of innovation, this study treats social media engagement as a crucial indicator for evaluating the effectiveness of destination image projection, revealing the impact of destination image projection on social media engagement patterns. Regarding methodological innovation, by using Bayesian network models and influence diagrams, this study intuitively illustrates the relationships and strengths of influence among various factors. It predicts the effects of different destination image projection strategies through scenario simulations.

First, official microblogs of universities trigger social media engagement following a power-law distribution. In other words, only a small number of microblogs lead to extensive social media engagement behaviors. Among the six dimensions of the Forbidden City’s destination image projection, scenarios with the highest likelihood of generating high social media engagement occur in microblogs related to the dimension of school natural landscapes. Previous research has found that popular topics such as the cherry blossoms of Wuhan University, Weiming Lake of Peking University, and Shui Mu Qing Hua of Tsinghua University are key to the transformation of destination images [39]. Similarly, this study also found that, compared to the dimension of school natural landscapes and school cultural and creative products, microblogs related to the dimension of school history and culture are less likely to generate extensive social media engagement. The stimulating effect of university destination image projection seems to be limited to attracting visual landscape attention and has not yet delved into the core charm of university history and culture.

Second, social media engagement is not only related to the dimensions of destination image projection but also to the interactive strategies, emotional representations, and publishing time frames in the content. Previous research mainly focused on comparing the differences between information published by social media users and destination marketing organizations, treating closing the gap between projected images and perceived images as the marketing goal [41]. In contrast, this study concentrates on the social media engagement triggered by destination image projection, emphasizing the role of university destination image dimensions and marketing strategies in shaping the interaction between social media users and destinations. This study finds that using interactive strategies in microblog texts can enhance social media engagement. Mentioning others and consumption guidance can attract more likes and forwards, while contribution guidance and creation guidance are more conducive to generating high comments. The use of positive emotional vocabulary not only helps shape a positive destination emotional image but also attracts more users to comment and like.

Third, the time frame for publishing microblogs is an essential decision factor for destination image projection. In different scenarios of university destination image projection, the optimal publishing time for triggering social media engagement varies. In the summer enrollment season, social media users are more likely to engage frequently with official university microblogs. Therefore, the summer vacation is a critical period for shaping the destination image and promoting enrollment intentions. The time frame of 12:00–22:59 is generally more likely to achieve higher social media engagement and is more likely to create microblogs with high comments and likes. Official university microblogs posted during active hours align with the active times of social media users, which is consistent with previous research [41]. Furthermore, this study demonstrates that different combinations of microblog content, interactive strategies, and publishing times can guide different types of social media engagement. Using an influence diagram, this study can simulate and infer the scale of social media engagement that can be achieved under different scenarios.

Finally, for historical and cultural dimension microblogs, which have the largest number but have not triggered extensive social media engagement, the combination of interactive strategies and time frames plays a crucial role in enhancing social media engagement. In the summer, guiding the public to engage in student enrollment behavior in the virtual space and organizing public-school cooperation activities in the spring is the optimal strategy for generating high social media engagement. Moreover, the time frames of 12:00–13:59 and 18:00–22:59 are the optimal publishing times for microblogs related to the school history and culture dimension. During these times, combining interactive guidance strategies can achieve more extensive social media engagement. Unlike other dimensions, mentioning others in microblogs related to the school history and culture dimension has a weaker impact on social media engagement. Only mentioning others in the context of enrollment promotion can promote social media engagement. This may be related to the special characteristics of the school’s history and cultural dimension. Compared to the popularity of celebrities, the matching degree between celebrities and the destination may be an essential factor affecting tourists’ attitudes toward the destination. However, how to stimulate the public’s cultural participation and cultural learning through interactive marketing is still an issue that needs further exploration.

### 5.2 Limitations and outlook

This study uses microblog data from official university accounts for the years 2018–2023 to construct Bayesian models and influence diagrams for social media engagement triggered by university destination image projection. Considering the diversity of tourism destinations, future research can further verify the models in other types of tourism destinations. In addition, this study employs quantitative analysis and model simulations to establish a model for the rules of university destination image projection that triggers social media engagement. However, it has not yet revealed the psychological and behavioral logic behind the model from the perspective of individual public users. Subsequent research still needs to use methods such as interviews and experiments to further investigate the mechanisms of university destination image network marketing.

## Supporting information

S1 Data (XLSX)

S2 Data (XLSX)
